# Living with the Trickster: Crows, Ravens, and Human Culture

**DOI:** 10.1371/journal.pbio.0040014

**Published:** 2006-01-17

**Authors:** Jackie Chappell

## Abstract

*In the Company of Crows and Ravens* traces the relationship between man and one group of feathered friends.

[Fig pbio-0040014-g001]

**Figure pbio-0040014-g001:**
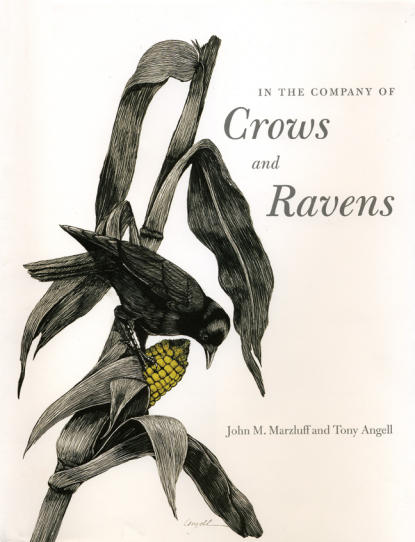
Marzluff JM, Angell T (2005) In the company of crows and ravens. Yale University Press. 408 p. ISBN (hardcover) 0-300-10076-0. US$19.80

Few groups of wild animals inspire such extreme opinions in the humans who observe them than members of the genus *Corvus*. In the book *In the Company of Crows and Ravens* [1], John Marzluff and Tony Angell quote Reverend Henry Ward Beecher's admiring words, “If men had wings and bore black feathers, few of them would be clever enough to be crows” (page 80 in [1]), but they also recount the opinion of their neighbour who sees crows as noisy, destructive, dirty, aggressive, and clever. Cleverness, it seems, is the only corvine attribute on which people agree.

People are rarely indifferent to crows, and this book explores the changes in opinion throughout our history of interactions with them. The authors argue—quite persuasively—that as well as affecting the biology and cultural evolution of crows, this relationship has had a significant influence on our own cultural evolution. They even suggest that there are instances of cultural coevolution between humans and crows. Perhaps such an intertwined history to some extent explains our strong feelings about crows.

The book covers an enormous amount of ground, documenting in an engaging way both the current research on ecology, social behaviour, and cognitive and communicative abilities of crows and their diverse representations in our legends, art, literature, and spiritual rituals. Consider the similarities between humans and crows: we are both highly social species, living mainly in small family groups but assembling in much larger numbers around rich resources. We are both intelligent, and adapt relatively easily to changes in environmental conditions. We are both generalists and opportunists about food, and can exploit a huge variety of resources. These similarities mean that for a large proportion of human history, crows have been a ubiquitous and prominent part of our world.

Our early interactions with crows (particularly ravens) seem to have resulted in a generally respectful—even awed—attitude towards them. Inuit legends describe how Crow brought light to the far north for his people, and the Norse god Odin was informed about the world by his two ravens, Hugin and Munin (Thought and Memory). Certainly, representing the thought and memory of a god would seem to be a fairly prestigious position. However, when humans became largely agrarian, crows became our competitors—stealing food and raiding crops—and had to be scared off with “scarecrows.” Later still, crows came to be associated with disease and death as they scavenged the corpses of the victims of the plague or of war, and for that reason, they are a convenient symbol of evil and of death in horror literature and films, to this day. In modern times, some species of crows have followed us into our cities, and their populations have boomed because they have exploited plentiful food resources, such as refuse. Once again, their adaptability brings them into conflict with humans, who have to devise ingenious methods to keep the crows out of their waste.

One odd phenomenon that surfaces repeatedly is that crows are commonly seen across cultures as tricksters, liars, and mischievous thieves. A reputation as a thief can be traced to their habit of stealing and caching small objects, but how can a nonlinguistic animal be a liar? It does, however, seem to help to have been a thief in order to catch a thief. Research by Emery and Clayton [2] showed that scrub jays (Aphelocoma coerulescens) with experience pilfering another jay's food caches moved their own caches to a new location, but only when they had been observed storing their food by another jay. Perhaps humans somehow recognise crows' intelligence, cooperation, deviousness, and sociality, and see a kind of reflection of themselves. Perhaps this might also help to explain the extreme reactions to crows; crows can change quickly from being friends and companions to competitors, opponents, and enemies (just like other humans) when we perceive that they might pose a threat to our security, food resources, or well-being.

How can a nonlinguistic animal be a liar?

The authors imply that our tangled, interwoven relationship with crows is unique. Although we do, for example, have a very close relationship with horses, and they have certainly influenced our cultural development, it is difficult to think of another nondomesticated animal with which we have such rich and varied relationships. Rats have played a large role in our history (most notably as accidental vectors of bubonic plague), but I struggle to think of any legends revering rats (or even mentioning them). Wolves and eagles often appear in our art, literature, and legends, but today most urbanised people have no contact at all with these animals themselves. So is it something about crows themselves that encourages such a unique relationship, or is it just chance that our cultural paths have crossed in this way?

Marzluff and Angell put forward the intriguing but controversial hypothesis that early interactions with crows and ravens as hunter–gatherers might have shaped our own evolutionary history. They argue that the need to defend our kills against scavenging crows might have promoted human cooperation and group living, which in turn would have pre-equipped us to deal with large mammalian predators and scavengers. They also mention a study by Vucetich and colleagues [3], which suggests that wolves might also have formed large social groups in order to defend their kills from ravens. They incur reduced foraging success because of intragroup interference, but gain more from being able to save their kills from the raven's beak. In a clever little twist, Marzluff and Angell suggest that the human/wolf association might have started with our shared interest in opposing the raven.

This book has a lot to offer. The illustrations by Angell are beautiful, and give the book a very special feel. It is engagingly written for a nonscientific audience, but endnotes and full references are there for those who are interested and would like to read further into the subject. Readers are also encouraged to observe crows for themselves and report their findings. One minor criticism is that some topics are covered in too little detail for the enthusiast, and also there is a heavy concentration on ravens (Corvus corax) and American crows (Corvus brachyrhynchos) at the expense of other species. However, these are an inevitable consequence of trying to cover such an enormous subject in a book suitable for nonscientific readers. Overall, *In the Company of Crows and Ravens* is highly recommended for the crow-fan and crow-hater alike. I find it interesting that just as scientists are beginning to probe the cognitive abilities of crows and are finding that they are much more impressive than we might have suspected, we are reminded that people seem to have known (but forgotten) how smart crows are all along.
